# Painless swelling of the forefoot and recurrent subcutaneous abscesses of the lower leg—Two distinct presentations illustrating the spectrum of eumycetoma in a nonendemic country

**DOI:** 10.1371/journal.pntd.0005360

**Published:** 2017-04-13

**Authors:** Adrian Schibli, Daniel Goldenberger, Andreas Krieg, Anna Hirschmann, Elisabeth Bruder, Michael Osthoff

**Affiliations:** 1 Division of Infectious Diseases, Triemli Hospital, Zürich, Switzerland; 2 Division of Clinical Microbiology, University Hospital Basel, Basel, Switzerland; 3 Division of Orthopedics, University Children's Hospital Basel, Basel, Switzerland; 4 Clinic of Radiology and Nuclear Medicine, University Hospital Basel, Basel, Switzerland; 5 Pathology, University Hospital Basel, Basel, Switzerland; 6 Division of Infectious Diseases & Hospital Epidemiology, University Hospital Basel, Basel, Switzerland; 7 Department of Biomedicine, University of Basel, Basel, Switzerland; University of California San Diego School of Medicine, UNITED STATES

## Introduction

Eumycetoma is a neglected tropical disease that is characterized by chronic progressive local inflammation of subcutaneous tissues with sinus formation and purulent discharge. Although the infection evolves from a small subcutaneous nodule, patients often present late with advanced disease, including destruction of surrounding tissue and subsequently loss of function. We report two cases of eumycetoma, one early and one late presentation. Written consent for publication was obtained from both patients.

## Case presentations

**Fig 1:**
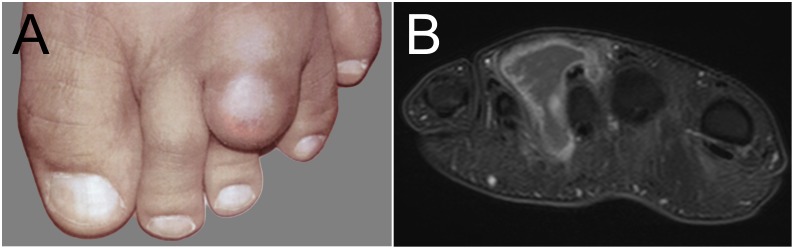
Case 1. Clinical presentation (A) before surgery: soft tissue swelling of the left forefoot. (B) T1-weighted fat-saturated post contrast MRI reveals a large polycyclic intermetatarsal mass surrounded by a thick wall.

Case 1: A previously healthy 41-year-old man was referred to our institution in November 2013 for the evaluation of a progressive swelling on his left forefoot (Fig 1A). The lesion was painless and had increased considerably in size in the last three months. The patient denied any constitutional symptoms. He had emigrated from India to Switzerland in 1996, and his last visit to the Indian subcontinent (Pakistan) dated back to 2008. Clinically, the lesion had a firm consistency with distal fluctuation but intact skin. Routine laboratory tests were unremarkable. Magnetic resonance imaging (MRI) of the left foot showed an interdigital mass of 25 x 27 x 43 mm extending from the dorsum of the foot towards the second and third interdigital space with marked capsular contrast enhancement. The lesion demonstrated a hyperintense signal on fat-saturated T2-weighted images with multiple punctuated low signal intensities within the lesion (Fig 1B). No bone erosion was evident. Based on these findings, a preliminary diagnosis of a soft tissue tumor was made, and surgical resection of the mass was planned. At the time of surgery, the overlying skin showed evidence of imminent perforation with evacuation of purulent liquid and particles of black granular texture after incision. The mass was resected in toto and sent for pathology investigations, suspecting a neoplastic origin.

**Fig 2:**
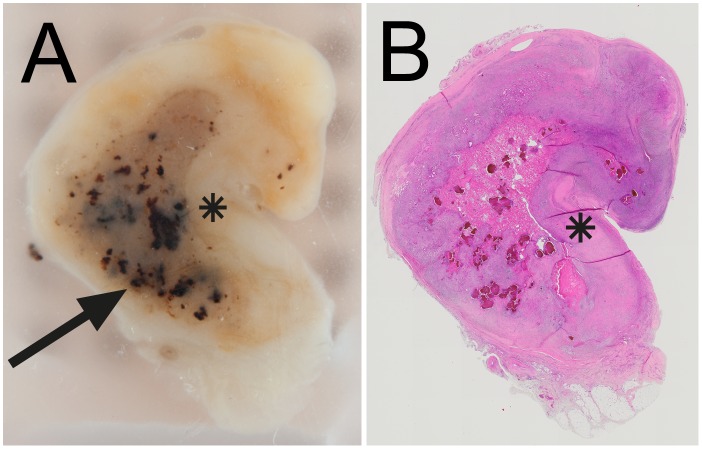
Case 1. (A) Macroscopic aspect of a paraffin block revealing multiple black granules in an oval zone of necrosis (arrow), surrounded by a wall of granulation tissue (asterisk). (B) Histological wholemount section with wall of fibrous tissue (asterisk) (Hematoxylin and eosin stain).

**Fig 3:**
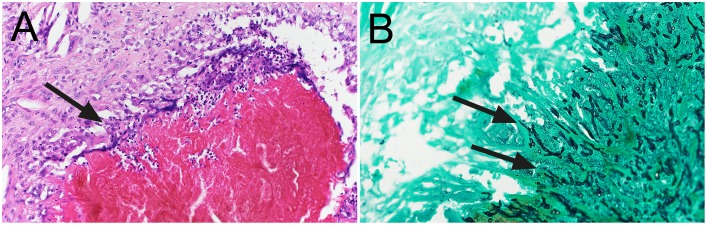
Case 1. High-power magnification. (A) Necrotizing inflammation with epithelioid-giant cells (arrow) (Hematoxylin and eosin stain). (B) Fungal hyphae (double arrow) (*Grocott's* Silver stain).

The resected mass measured 20 x 25 x 41 mm and was surrounded by a pseudocapsule. Cross-sections revealed macroscopic fragments of black grains (Fig 2) and an inflammatory reaction characterized by areas of focal necrosis and epithelioid and giant cells surrounding fungal hyphae staining positive for Grocott (Fig 3).

After surgery, the patient was sent to the outpatient infectious diseases clinic for further evaluation. Taking into account the patient’s travel history, presentation, and histopathology results, a differential diagnosis of eumycetoma was entertained, and panfungal polymerase chain reaction (PCR) targeting the internal transcribed spacer (ITS) regions 1 and 2 of the formalin-fixed paraffin-embedded tissue was ordered [1]. Sequence analysis of both ITS1 and ITS2 (587 nucleotides) revealed a 100% match with reference sequences of *Madurella mycetomatis*. Hence, a diagnosis of eumycetoma caused by *M*. *mycetomatis* was made, and treatment with 100 mg of itraconazole twice daily was initiated with regular therapeutic drug monitoring. Four months after surgery, there were no signs of recurrent soft tissue swelling or osteomyelitis on a repeated MRI scan. The patient was treated for six months without evidence of relapse 30 months after cessation of treatment.

**Fig 4:**
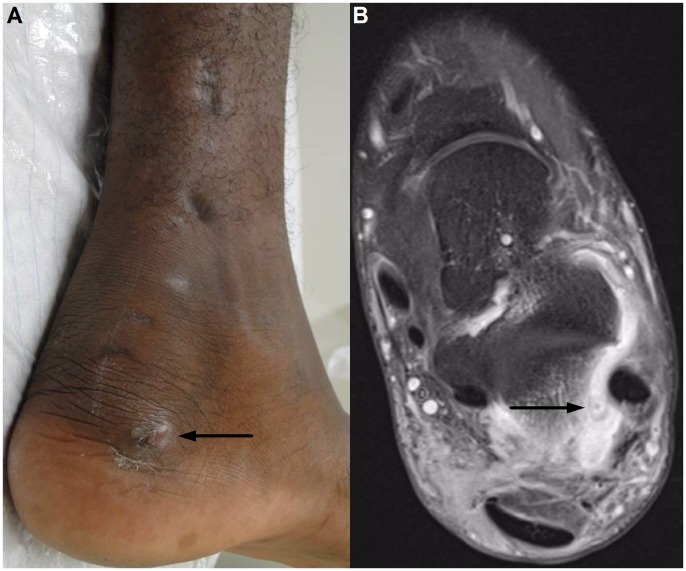
Case 2. (A) Clinical presentation after drainage of the subcutaneous abscess (arrow). (B) T2 fat-saturated MRI reveals diffuse inflammation of the soft tissue and a pathognomonic central hypointense dot (arrow) surrounded by hyperintensities reflecting the grain.

Case 2: A 21-year-old Eritrean migrant presented to our emergency department with progressive pain and swelling of his left ankle. On examination, a painful fluctuation was noted below his medial ankle (Fig 4A) consistent with a subcutaneous abscess. In addition, multiple scars were evident on his left lower leg. Routine laboratory tests were unremarkable. Subsequently, the abscess was drained and sent for standard culture. A seven-day course of amoxicillin/clavulanic acid was prescribed.

The patient was born in Sudan but had lived in Eritrea since the age of four. After leaving Eritrea about four months earlier with stopovers in Sudan (two months) and Libya (five weeks), he arrived in Italy two weeks before presenting to our hospital. He reported recurrent local infections on his left foot since the age of 10, which had been treated with local dressings only.

**Fig 5:**
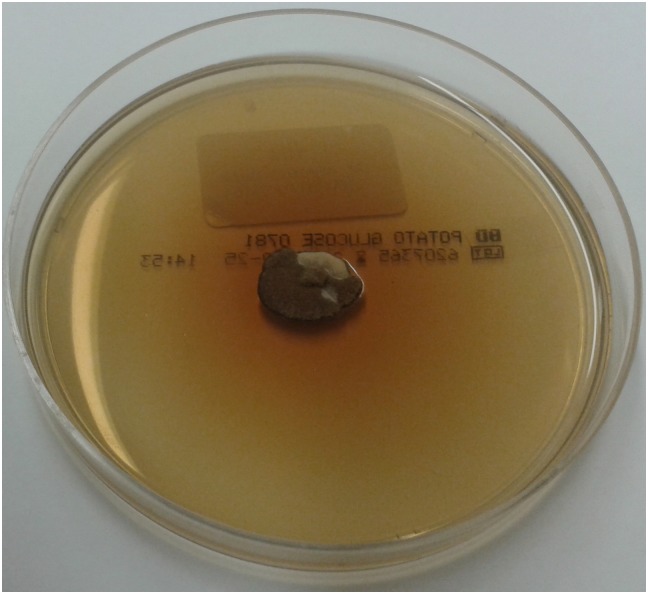
Case 2. Agar plate of the *M*. *mycetomatis* isolate producing a brownish diffusible pigment in the agar.

Interestingly, abscess cultures flagged positive with a mold, whereas bacterial growth was absent (Fig 5). Finally, the mold was identified as *M*. *mycetomatis* using panfungal PCR and sequence analysis, as described above. Resistance testing indicated susceptibility to amphotericin B, itraconazole (minimum inhibitory concentration of 0.003 mg/l), posaconazole, and voriconazole.

When the patient was followed up in our outpatient infectious diseases clinic, he reported ongoing pain in his left ankle. An MRI ordered to characterize the extent of the disease demonstrated residual infection with the pathognomonic appearance of round hyperintensities with a central hypointense dot (Fig 4B), reflecting the grain. Adjacent inflammation of the subtalar joint was noted. Treatment with 100 mg of itraconazole twice daily was initiated, which resulted in a marked reduction of pain after four weeks. We had intended to continue medical treatment for six months and to repeat the MRI scan in order to decide about surgical debridement/resection. Unfortunately, the patient was transferred back to Italy and was lost to follow-up.

## Case discussion

*M*. *mycetomatis* is the most prevalent causative agent of eumycetoma, a chronic fungal infection of the skin and soft tissue. The feet are affected most frequently, supporting the hypothesis of infection by local implantation of the organism. Its bacterial counterpart is actinomycetoma, which is most commonly caused by *Streptomyces somaliensis* and *Nocardia brasiliensis* [2, 3].

**Fig 6:**
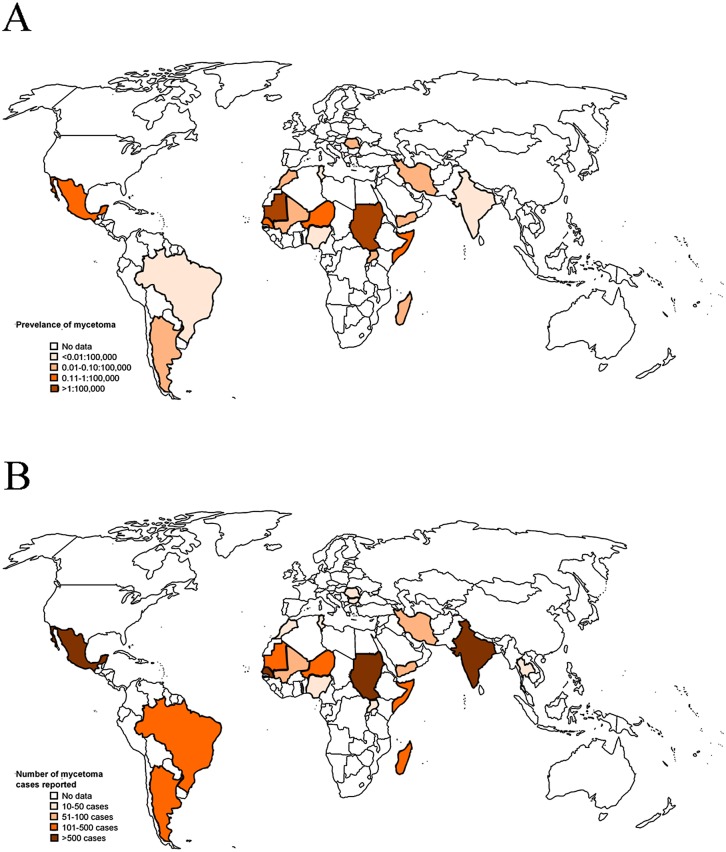
Prevalence and number of reported cases of mycetoma. (A) Average prevalence of mycetoma cases as calculated by the number of cases reported in a year in a certain country divided by the total population of that country of that same year as reported by www.indexmundi.com/facts/indicators/SP.POP.TOTL/compare. (B) The average number of mycetoma cases reported per year per country. Reprinted from van de Sande [4] under the terms of the Creative Commons Attribution License (http://dx.doi.org/10.1371/journal.pntd.0002550.g002).

Eumycetoma due to *M*. *mycetomatis* is endemic in tropical and subtropical countries with the highest prevalence in Africa (Sudan) and the Indian subcontinent, whereas actinomycetoma is more common in Central and South America (Fig 6) [4]. The initial presentation is a slowly progressive and painless subcutaneous swelling. In contrast to endemic areas, where people tend to present rather late (similar to patient 2), the diagnosis in patient 1 was established without substantial delay, although eumycetoma was not included in the differential diagnosis before surgery. The triad of a painless swelling, macroscopically visible dark grains, and the patient’s origin from an endemic country as well as histological demonstration of fungal hyphae raised our suspicion of eumycetoma. Identification of *M*. *mycetomatis* on ITS sequencing confirmed the diagnosis [5]. The presentation of the second patient was more classical, with recurrent soft tissue infections in the past and an abscess with a draining sinus on his left foot.

Despite complete resection, we opted for additional antifungal treatment in patient 1. Due to high relapse rates after surgical monotherapy, only small and well-encapsulated areas can be cured by resection. In general, early diagnosis and adequate antifungal treatment for many months followed by wide surgical excision appear to be the most important steps for a successful outcome. In case of large lesions, surgery can be performed adjunctively after months of antifungal therapy. Importantly, follow-up (including radiological examinations such as MRI or ultrasound) should continue after cessation of antifungal treatment, as relapses may occur. Oral itraconazole is the preferred antifungal treatment, whereas ketoconazole is no longer an option due to serious side effects [6]. Newer generation triazoles have been used successfully [7] and have demonstrated excellent activity in vitro [8], whereas *M*. *mycetomatis* is usually resistant to echinocandins [9]. In general, antifungal treatment alone is rarely curative but reduces the size of the lesions and enables less extensive surgical debridement.

The present cases underscore the importance of considering epidemiological clues for eumycetoma in the differential diagnosis of painless soft tissue swellings or recurrent subcutaneous abscesses of the feet. Physicians in countries currently hosting refugees from tropical areas of Africa, Asia, and Central and South America need a high index of suspicion for this infection, which is uncommon in many parts of the world, including Europe. Additionally, molecular identification by panfungal PCR is a powerful tool to identify the causative agent, in particular if identification by culture is not possible.

**Key learning points**

- This presentation is a useful reminder for clinicians to consider epidemiological clues for eumycetoma in the differential diagnosis of painless soft tissue swellings or recurrent subcutaneous abscesses, in particular of the lower leg.
- Clinicians in countries currently hosting refugees from tropical areas of Africa, Asia, and Central and South America need a high index of suspicion for this uncommon infection.
- Panfungal PCR may serve as a powerful tool to identify the causative agent of eumycetoma, in particular if culture is not available.
- Treatment of eumycetoma necessitates an interdisciplinary approach, with surgeons, radiologists, and infectious diseases physicians being involved.
